# Supplementary material for: Segmented readout for Cherenkov time-of-flight positron emission tomography detectors based on bismuth germanate
Source: ArXiv. 2024 Oct 16:arXiv:2410.12161v1. Preprint. [Version 1] (PMC11527102)
Supplement: Supplement 1 [file NIHPP2410.12161v1-supplement-1.pdf]

Supplementary Table 1. Coincidence timing resolution values measured using different BGO crystal lengths.

Length	5 mm		10 mm		15 mm		20 mm	
[ps]	FWHM	FWTM	FWHM	FWTM	FWHM	FWTM	FWHM	FWTM
T_A	185 ± 3	826 ± 36	210 ± 11	1084 ± 56	230 ± 12	1200 ± 70	255 ± 14	1257 ± 53
T_B	189 ± 4	861 ± 43	203 ± 13	1005 ± 22	224 ± 8	1131 ± 45	250 ± 13	1277 ± 44
T_{Early} All Events	167 ± 3	423 ± 27	175 ± 5	502 ± 17	186 ± 5	590 ± 22	204 ± 5	648 ± 28
T_{Early} ($\Delta T_k = 300\ ps$)	161 ± 6	359 ± 11	164 ± 4	399 ± 16	183 ± 8	500 ± 28	181 ± 10	500 ± 20

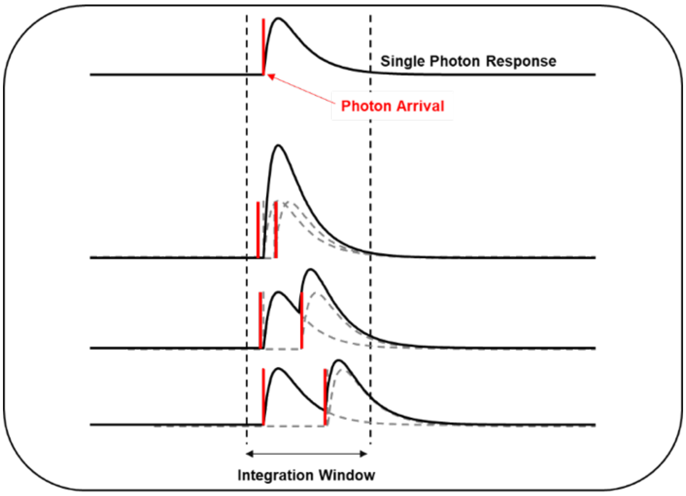

Supplementary Figure 1. The effect of photon arrival delay on the integration value.

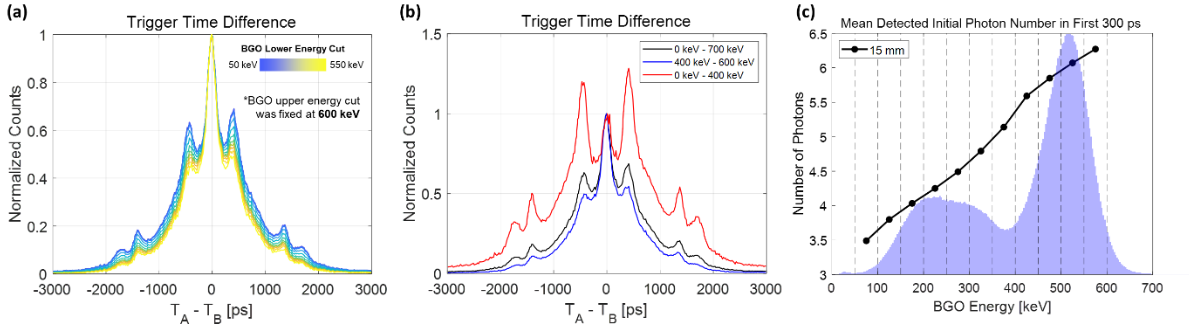

Supplementary Figure 2. Distributions of trigger time differences with varying energy lower limits. (a) With a fixed 600 keV upper limit (b) Photopeak (400 – 700 keV) and scatter (0 – 400 keV) energy regions. (c) The number of initial photons for different energy intervals (50 – 100 keV, 100 – 150 keV, …, 550 – 600 keV).

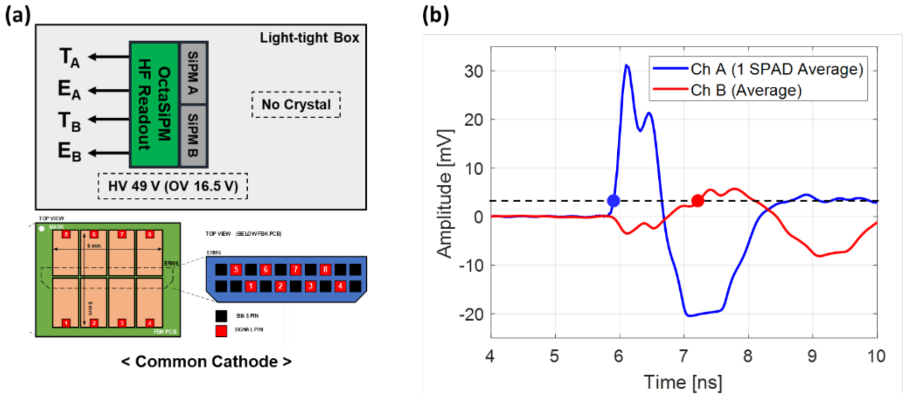

Supplementary Figure 3. Electronic crosstalk due to the common cathode configuration.

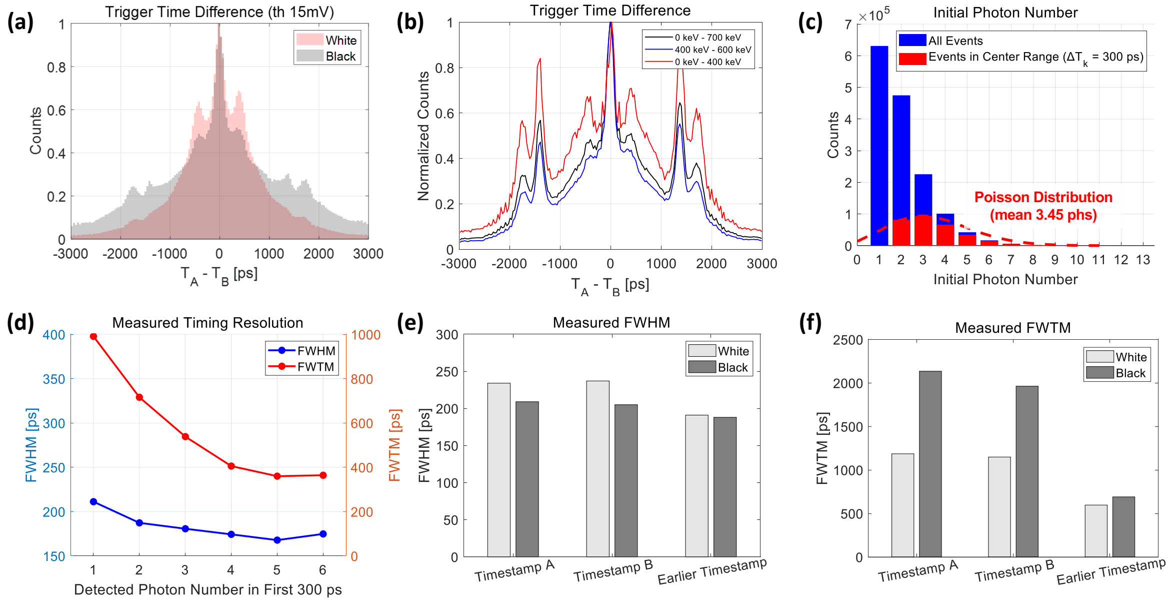

Supplementary Figure 4. Black reflector results.

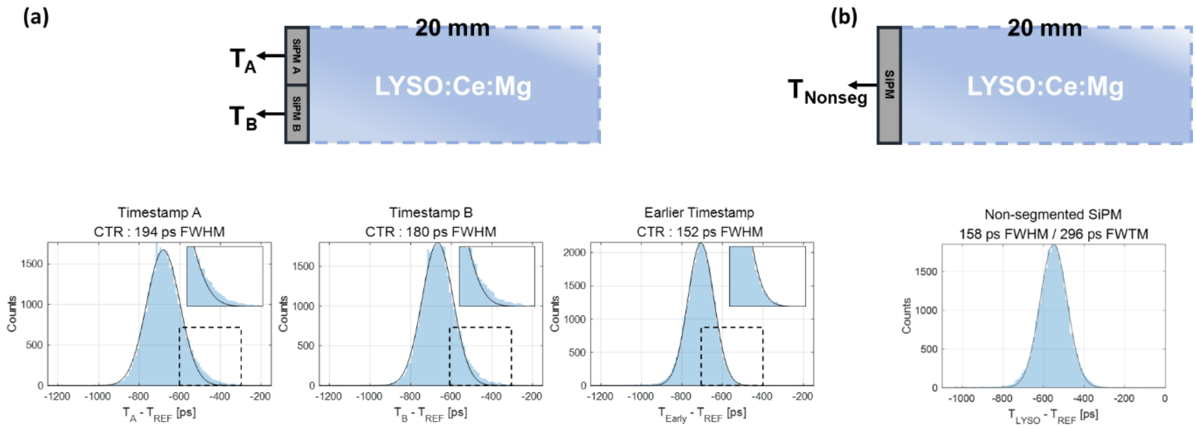

Supplementary Figure 5. Measurements from LYSO:(Ce,Mg) coupled to an OctaSiPM (a) and a non-segmented SiPM (b).
